# 

**Published:** 1878-02

**Authors:** 


### CONTENTS:

ARTICLE I.—Annual Address of the President of the Virginia State Dental Association........ 433

ARTICLE II.—Annual Meeting of the Virginia State Dental Association. (Continued.)........ 443

ARTICLE III—Jottings in Operative Dentistry. By Jas. F. Thompson., D. D. S..... 459

ARTICLE IV.—Pulp Capping Again. By Dr. W. C. Barrett........ 464

ARTICLE V.—Chancre of the Lip—Was it Communicated by a Dentist's Instruments ?........ 468

ARTICLE VI.—Cause of Dental Caries........ 471

### EDITORIAL, ETC.

Professor Henry Reginald Noel........ 472
Neuralgia Treated with Blue Glass........ 474

### BIBLIOGRAPHICAL.

Vick's Illustrated Catalogue and Floral Guide, 1878........ 475

Music Publications........ 475

Contribution to the History of Medical Education and Medical Institution in the United States of America, 1776–1876........ 476

Wine in the Different Forms of Anœmia and Atonic Gout........ 476

A Review of the Recent Theories of Nerve Action, the Use of Electricity in Medicine and Surgery, &c........ 476

### MONTHLY SUMMARY.

Plating of Iron and Steel with Nickel and Cobalt by Immersion........ 476

Death from Chloroform Averted by the Inhalation of Nitrite of Amyl........ 477

Alumni Meeting........ 479

Repeated Excision of the Inferior Dental and Gustatory Nerves for the Cure of Dental Neuralgia........ 479

Death from Inhalation of Ether........ 479

Nitric Acid for Hoarseness........ 480

